# A plasmonic route for the integrated wireless communication of subdiffraction-limited signals

**DOI:** 10.1038/s41377-020-00355-y

**Published:** 2020-07-02

**Authors:** Hao Chi Zhang, Le Peng Zhang, Pei Hang He, Jie Xu, Cheng Qian, Francisco J. Garcia-Vidal, Tie Jun Cui

**Affiliations:** 1 State Key Laboratory of Millimeter Waves, Southeast University, 210096 Nanjing, China; 2 Departamento de Física Teórica de la Materia Condensada and Condensed Matter Physics Center (IFIMAC), Universidad Autónoma de Madrid, Madrid, Spain; 3 Donostia International Physics Center (DIPC), Donostia/San Sebastian, Spain

**Keywords:** Physics, Optical physics

## Abstract

Perfect lenses, superlenses and time-reversal mirrors can support and spatially separate evanescent waves, which is the basis for detecting subwavelength information in the far field. However, the inherent limitations of these methods have prevented the development of systems to dynamically distinguish subdiffraction-limited signals. Utilizing the physical merits of spoof surface plasmon polaritons (SPPs), we demonstrate that subdiffraction-limited signals can be transmitted on planar integrated SPP channels with low loss, low channel interference, and high gain and can be radiated with a very low environmental sensitivity. Furthermore, we show how deep subdiffraction-limited signals that are spatially coupled can be distinguished after line-of-sight wireless transmission. For a visualized demonstration, we realize the high-quality wireless communication of two movies on subwavelength channels over the line of sight in real time using our plasmonic scheme, showing significant advantages over the conventional methods.

## Surface plasmons open new channels for communication

The unique properties of surface plasmons enable wireless transmission of signals separated by less than one wavelength. Until recently, it was considered impossible to distinguish signals with sub-wavelength separation, due to the so-called diffraction limit. This limit can be overcome using artificial structures called metamaterials, but it is difficult to integrate these new components with conventional electronics. Now, Tie Jun Cui at Southeast University in Nanjing, China, and co-workers have shown that sub-wavelength signals can be transmitted using surface plasmon polaritons (SPPs)—combinations of electromagnetic waves and charge motion that travel on the surface of a metal. By encoding signals in ‘spoof’ SPPs that mimic natural SPPs, the team were able to wirelessly transmit two high-definition movies on channels just one-tenth of a wavelength apart, even with a concrete wall in the way.

## Introduction

Distinguishing two or more signals with subwavelength separation has accelerated research in many fields, including photonics, super-resolution imaging, and dense communication, which are always hampered by the so-called diffraction limit. Subdiffraction-limited information from an object is carried by evanescent waves and decays exponentially before reaching the far field. Although many methods, including far-field time-reversal mirrors^1–3^ and optical diffraction tomography setups^4,5^, have been proposed to break the diffraction limit, these schemes work at the price of slow transmission speed, which makes them inefficient for real-time applications.

Moreover, several designs of metamaterials^6–8^ with novel electromagnetic (EM) properties realized by artificial metallic structures have been used for both super-resolution imaging and signal transmission to directly distinguish two or more subdiffraction-limited signals. Among them, *metalenses* such as hyperlenses and superlenses^9–12^ have been widely studied to realize the real-time far-field imaging of subwavelength objects. In fact, those lens designs can be used in line-of-sight communication systems, similar to time-reversal mirrors^1^. However, evanescent subdiffraction-limited information has to be captured in the near field of a lens, and thus subdiffraction-limit technology based on this strategy can be applied only on the surface of an object. As a consequence, these bulky lenses hinder the realization of conformal systems. More importantly, inherent loss and strong sensitivity to the environment that leads to channel interference limit the application of metalenses in integrated wireless communication systems.

In contrast to the speed and size limitations of the abovementioned methods, real application scenarios always require robust, flexible, and dynamic far-field transmissions of subdiffraction-limit information, for instance, in the highly dense multiple input multiple output system^13–15^. One of the possible approaches is to use localized channels to guide subdiffraction-limited signals to radiation antennas with orthogonal features, for example, with orthogonal polaritons. However, for mature circuit technologies (such as integrated semiconductor chips and wireless communication systems), the main problem is that traditional microstrip-based circuits cannot support the transmission of subdiffraction-limit information, i.e., they cannot distinguish signals with subwavelength separation. Spoof surface plasmon polaritons (SPPs) make it possible to break the barrier between subdiffraction-limit signal communication and integrated circuit technologies. As an alternative waveguide to microstrips, low-frequency spoof SPPs that mimic the properties of natural SPPs in the optical regime have shown promising potential for many advanced applications owing to their very deep subwavelength confinement but moderate propagation losses^16–21^. In particular, one-dimensional ultrathin corrugated metallic strips have been shown to be efficient *metawaveguides* for transmitting spoof SPP modes on planar circuits^22–44^, which can be utilized to solve the crosstalk problem in dense communications where compactly arranged channels possess features similar to those of the subwavelength channels. As a result, it has been recently demonstrated that these types of metawaveguides are efficient carriers of deep subdiffraction-limited EM signals and are able to largely overcome the diffraction limit for propagating EM waves. In addition, thanks to their ultrathin and flexible characteristics, these metawaveguides can adapt conformally to the surface geometry of an underlying object.

Here we present a compact planar wireless communication system with integrated SPP channels to realize the integrated wireless communication of subdiffraction-limited signals. Two separate modulated digital signals, which can also be called intermediate frequency (IF) signals, are independently transmitted on two subdiffraction-limited EM channels with a separation smaller than one-tenth of the wavelength (the center spacing of the channels is approximately a quarter of the wavelength), which can hardly be achieved by traditional shielded waveguides (e.g., the coaxial line and substrate integrated waveguide). We demonstrate that a series of inherent channel interference problems due to repeated coupling–gain–coupling processes in adjacent subdiffraction-limit channels can be solved by employing the unprecedented field confinement associated with the spoof SPP modes. These two subdiffraction-limited EM signals can travel along the integrated SPP channels with low transmission loss, low channel interference, and high gain and are then radiated to the far field with orthogonal polarizations, which empower the subdiffraction-limited signals with superior environmental resistance for line-of-sight wireless transmission. Meanwhile, the field enhancement of spoof SPP modes promises high demodulation power sensitivity to the receiver, designed as the inverse of the process conducted by the transmitter. These merits contribute to the realization of high-quality wireless communication of subdiffraction-limited EM signals. As a proof of concept, we demonstrate these capabilities visually by carrying out experiments of the simultaneous line-of-sight wireless communication of two high-definition movies by our plasmonic system, showing a much better performance than that achieved by using the conventional microstrip system. To the best of our knowledge, this work is the first systematic integration of spoof SPP technologies, demonstrating the potential to realize novel dense communication systems with flexible and dynamic far-field transmissions.

## Results

**Fig. 1:**
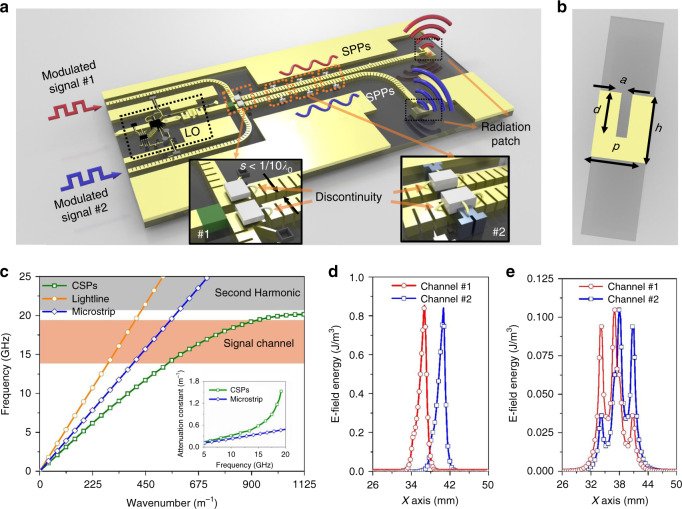
Schematic representation of the system, propagation characteristics, and subwavelength confinement of the SPP modes. **a** Schematic of the operation principle of the SPP-based system, which is composed of two independent signal-processing channels with a very deep subwavelength-scale separation and a shared continuous-wave oscillator. At the entrance of the device, two parallel IF signals are coupled into the system through an SPP connector, modulated with carrier waves using an SPP-based harmonic mixer, and amplified by a chain of SPP-based amplifiers, where the bonding wire connections bring significant discontinuities (inset). After being transported by the two metawaveguides, the subwavelength signals are radiated to free space by two antennas with orthogonal polarizations. Alternatively, the receiver is designed as the inverse of the transmitter. **b** Schematic of the SPP unit cell of the metawaveguides used in our design, which is fabricated in a single 18-µm-thick layer of copper (yellow) deposited on a 787-µm-thick dielectric layer (gray). The detailed geometrical parameters can be found in the “Methods” section. **c** The real (main panel) and imaginary (inset) components of the dispersion curves of the SPPs supported by the metawaveguides compared with those of the propagating EM modes of a microstrip. **d** The E-field energy distribution of the signal carried by the plasmonic metawaveguides on a test line that is 787 µm above the structured surface. **e** The E-field energy distribution of the signal carried by the microstrip system on the test line at the same location as in **d**

In the experimental set-up, both IF and radio frequency (RF) signals are transmitted in the spoof SPP modes (Fig. 1a). The metawaveguides, i.e., the ultrathin corrugated metallic strips, support the spoof SPP modes (Fig. 1a), the propagating characteristics (dispersion and field pattern) of which can be tuned by changing the geometrical parameters, for instance, the depth (*d*) and width (*a*) of the grooves on the metallic strip (Fig. 1b). The specific geometrical parameters of the metawaveguides used in our prototype are given in the “Methods” section.

The two modulated digital (IF) signals in the SPP modes are fed on two pure metawaveguides first and then input into two subwavelength-integrated SPP channels composed of metawaveguides and bare semiconductor chips. Subdiffraction-limited IF signals on the integrated SPP channel are mixed into RF signals by a shared wave produced by a local oscillator (LO) using two harmonic mixers first, which can be regarded as two effective sources packaged in subwavelength space (see inset #1 in Fig. 1a), and then are gained by a chain of amplifiers (see inset #2 in Fig. 1a) to increase the signal-to-noise ratio (SNR) in long-distance wireless communications (Fig. 1a). At the end of the integrated SPP channel, the two EM signals are radiated into free space for wireless communication by two antennas that have orthogonal polarizations. In this way, the two free-space EM waves can be retrieved by the other two antennas in the SPP receiver (not shown in Fig. 1a), which is just the inverse of the process carried out by the SPP transmitter.

To account for the fundamentals of the integrated SPP channel, we first analyze the dispersion relation (frequency versus wavenumber) associated with the spoof SPP mode supported by the metawaveguide and compare it with that of a traditional microstrip. The wavenumber of the SPP mode increases nonlinearly with frequency and is faster than that of the EM mode supported by the microstrip (Fig. 1c). Importantly, it approaches an asymptote at the cutoff frequency (20 GHz in this design), which is mainly dictated by the groove depth. We choose the operating frequency (18 GHz) to be near this cutoff frequency, where the SPP dispersion curve deviates significantly from the light line, resulting in a very strong field confinement. It is worth mentioning that this strong confinement results in a slightly larger attenuation constant than that of the microstrip (see inset of Fig. 1c), but it is acceptable for engineering applications. The strong field confinement associated with the propagating spoof SPP modes makes the overlap between EM signals carried by the two metawaveguides located at a deep subwavelength separation (*λ*/10) negligible (Fig. 1d). In contrast, the field energy distributions of the EM waves carried by two microstrips with the same width and separation present very strong coupling, such that the two EM signals cannot be distinguished (Fig. 1e).

Nevertheless, since the channels are composed of both continuous waveguides and integrated bare chips, the interference is much more complex and closer to practical application than pure waveguide coupling^45–47^ due to the repeated coupling–gain–coupling process. Signals coupled to the adjacent waveguide will be gained by amplifiers. The obtained coupling signal can be coupled back on the following waveguide, which could occur repeatedly on two subwavelength channels. Specifically, both leaky waves from the discontinuity and waveguide coupling contribute to the channel interference.

**Fig. 2:**
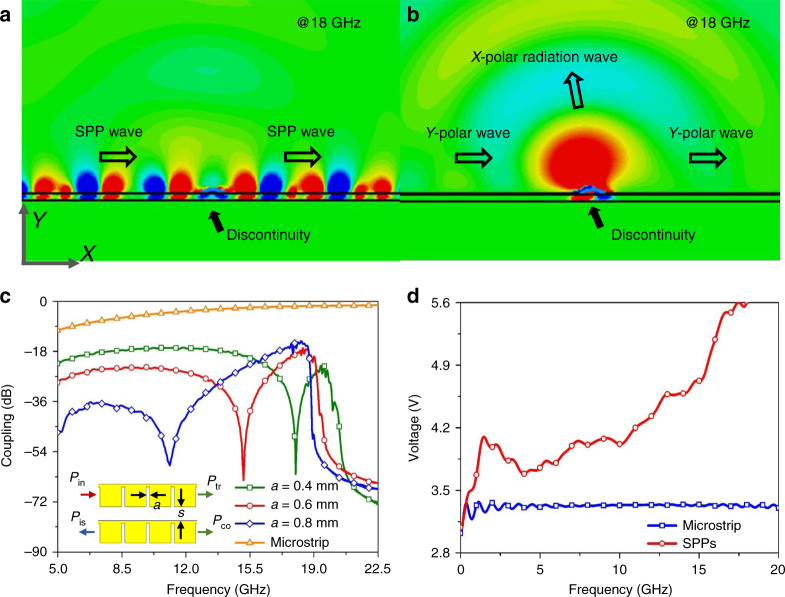
Superiorities of the SPP channels over the microstrip channels in this system framework. **a** The *x*-component of the electric-field distribution of the SPP mode propagating along the metawaveguide at 18 GHz when an inevitable discontinuity (bonding wire connection) is introduced. A negligible leaky wave can be observed. **b** The *x*-component of the electric-field distribution of the EM mode supported by the microstrip for the same setting as in **a**. Here the leaky waves to free space are clearly observed, which will impact adjacent channels and alias with the signal from the antennas. **c** The coupling of continuous parts of two subdiffraction-limit channels (separation *s* = 1.5 mm), in which the coupling is defined by the ratio of the coupling power, *P*_co_, to the incident power, *P*_in_. Owing to the subwavelength field confinement of SPPs, the continuous-part coupling of the SPP channels is much smaller than that of the microstrip channels. The spectral location of the frequency in which this coupling is minimal can be further tuned by the groove width, *a*. **d** Voltage differences between the strips and metallic ground under the same input power (0.23 W), which can help in constructing an SPP-based digital interface that displays higher power sensitivity for demodulation than achieved by microstrips

To integrate bare chips (e.g., the case sketched in the inset of Fig. 1a) onto the metawaveguide (both the corrugated metallic strips and the traditional microstrips), bonding gold wires are used to realize an electrical connection between them. The discontinuity of this connection can lead to leaky EM waves that would impact adjacent channels and alias with the signal from the antennas. However, the tightly confined SPP fields can dramatically reduce this radiation into free space (Fig. 2a) compared to the microstrip case (Fig. 2b), which can be attributed to inherent wavevector mismatching between the spoof SPP mode and leakage wave mode.

Although the waveguide coupling of the SPP mode is smaller than that of the microstrips due to the subwavelength confinement of the SPP fields, the waveguide coupling has to be further suppressed to an ultralow level. Note that the coupling between the metawaveguides is mainly controlled by the groove width *a* (Fig. 2c); hence, we can dramatically reduce the coupling by tuning this geometrical parameter. In this design, we choose *a* = 0.4 mm so that the coupling is minimal (<−50 dB) at the operating frequency. In contrast, the crosstalk of two microstrip channels is approximately −3 dB at 18 GHz, which means that approximately half of the signal power on Channel 1 will be coupled to Channel 2 and vice versa. Since signals on the two microstrip channels are at the same power level, the crosstalk behaves similar to serious noise.

In addition to suppressing the interference between channels utilizing the inherent field confinement of the SPP modes, metawaveguides promise high demodulation power sensitivity to the receiver based on their field enhancement property. Since the energy of the SPP mode is mainly concentrated near the corrugated side of the metawaveguide, the voltage difference between this area and the ground is effectively enhanced even though the transmission loss of the SPP metawaveguide is slightly higher than that of the microstrip. For an input power that is not large enough for a traditional microstrip (with the same width) to exceed the digital threshold of modulation, our SPP metawaveguide can instead provide a higher induced voltage to excite the demodulation devices (Fig. 2d). This capability will help significantly improve the power sensitivity of the receiver.

Both the low channel interference and high power sensitivity will increase the transmission distance attainable by the SPP-based system, thus making SPP metawaveguides suitable for long-distance and line-of-sight wireless communications of very deep subdiffraction-limited EM signals, as shown below.

**Fig. 3:**
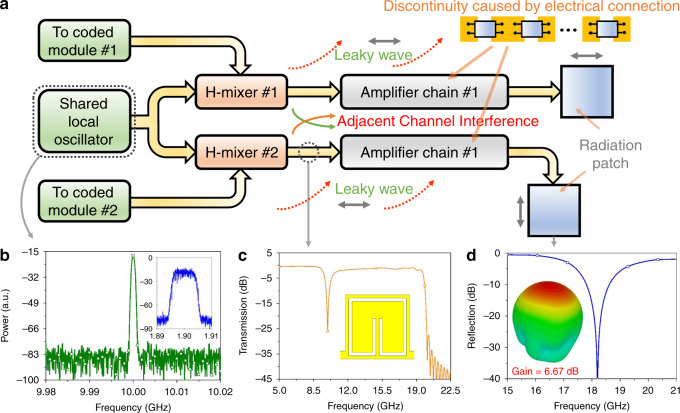
Schematic of the system framework and some core SPP devices for the newly proposed subdiffraction-limit transmission system. **a** System framework schematic of the SPP wireless communication system. By introducing spoof SPPs, a series of inherent signal interference problems in adjacent subdiffraction-limit channels, including leaky waves and continuous-part coupling, can be solved from the basic physical view without any extra cost. **b** The measured frequency spectrum of the shared local oscillator, which can spontaneously generate a continuous carrier wave at 10 GHz, and of the coded signal (inset). **c** The measured frequency spectrum and structure (inset) of a designed ultracompact filter, which can block the fundamental signal at approximately 10 GHz. **d** The measured frequency spectrum and the 3-D gain of the antenna, which can transmit signals in the designed frequency band with weak directivity and high efficiency

**Fig. 4:**
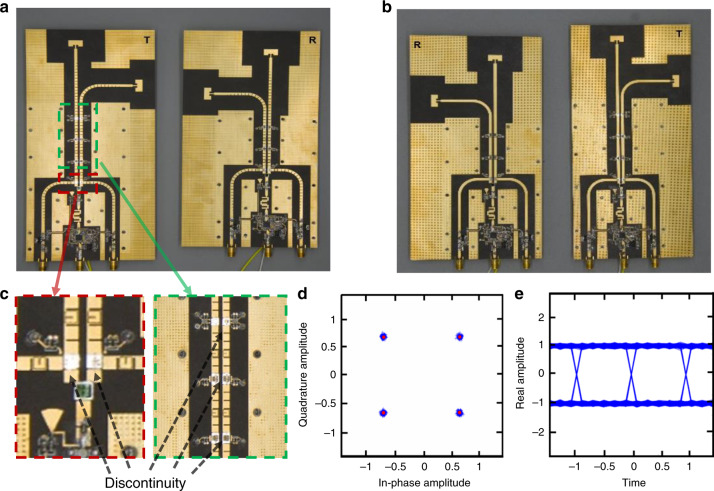
Photographs of the fabricated wireless communication system, and test results of the signal quality. **a** Photographs of the fabricated SPP wireless communication system. **b** Photographs of the fabricated microstrip wireless communication system. **c** Enlarged structure diagram of the discontinuity caused by the electrical connection. **d** Constellation maps measured in the SPP wireless communication system. **e** Eye patterns measured in the SPP wireless communication system

The SPP-based communication system was fabricated in a single-layer dielectric substrate. A detailed account of the different components of the wireless communication system is rendered in Fig. 3a and “Methods” section. A shared LO with an oscillation frequency of 10 GHz is used to provide the carrier EM waves for the IF signals (Fig. 3b). Two digital signals with spectral components of 1.9 GHz are modulated by the RF carrier waves through an SPP harmonic mixer (H-mixer), which includes the second harmonic and mixing generation. An ultracompact filter is designed and set before the amplification to block the fundamental signal at approximately 10 GHz (Fig. 3c). More importantly, the second harmonic of the fundamental signal at approximately 20 GHz and the up-conversion component at approximately 21.9 GHz are both cut off by the natural filtering feature of the SPP modes (Fig. 1c). After multistage amplifications of the SPP waves, the two subdiffraction-limited signals are radiated by two identically designed but orthogonally polarized wide-angle patch antennas with the radiation band near the operating frequency, 18 GHz, which possess weak directivity but high efficiency (see Fig. 3d). Owing to the isolation of cross polarizations, the two EM signals can be extracted in the SPP receiver separately after long-distance and line-of-sight wireless communications. A more detailed discussion on the design and performance of all the abovementioned passive and active SPP components is given in the “Methods” section. For comparison purposes, transmitter–receiver pairs based on the SPP metawaveguide (Fig. 4a) and traditional microstrips (Fig. 4b) with the same framework and the same size are fabricated. As commented before, the receiver is designed as the inverse version of the transmitter.

**Fig. 5:**
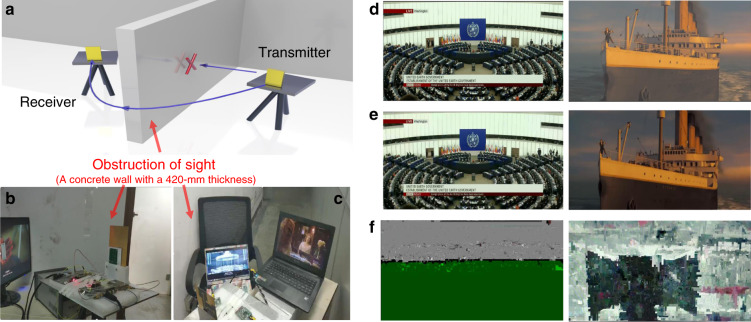
Test scenario schematic and line-of-sight real-time communication experimental results of the SPP and microstrip systems. In this experiment, two 4K movies (*The Wandering Earth* and *Titanic*) are transmitted via the two subdiffraction-limit channels. **a** Test scenario schematic of the line-of-sight real-time transmission, where the wireless signal emitted by the transmitter goes through the barrier and is received by the receiver. Photographs of the test scenario of receiver (**b**) and transmitter (**c**). **d** Frames of the two original movies to be transmitted. The same frames as decoded by the receiver after being transmitted by the SPP-based system (**e**) and by the microstrip system (**f**)

To test the feasibility of the SPP integrated wireless communication system quantitatively, line-of-sight real-time communication experiments of our prototype are performed in a very demanding scenario, in which a barrier is located between the transmitter and receiver to block the line of sight (Fig. 5a). A detailed description of the test scenario can be found in the “Methods” section. To overcome the barrier blockage, both the transmitter and receiver can be rotated; therefore, the free-space wireless signals can bypass the carrier to achieve line-of-sight communications. The real-time communication speed of the SPP system is >500 Mbps, and the bit error rate is <10^–5^ (see “Methods” for details regarding the method of measurement). The favorable constellation pattern (Fig. 4d) and eye pattern (Fig. 4e) of the measured signals in the plasmonic wireless communication system under line-of-sight transmission conditions (@ 24 °C) demonstrate the excellent communication quality.

To visualize the communication process of subdiffraction-limited signals, we select two 4K movies (*Titanic* and *The Wandering Earth*) as the simultaneous input signals of the two subwavelength channels (Fig. 5b) and test the real-time line-of-sight communications of the two movies (Fig. 5c). Four screens are connected to the two input ports and output ports to read the movie signals. The frames of the original two movies (Fig. 5d) transmitted by the transmitter are captured by the receiver and read by the screens. As shown in these figures, the frames received in the SPP-based system (Fig. 5d) are synchronized in both image quality and time with the original frames. In other words, the very high-quality and line-of-sight wireless communication of two subwavelength-channel movies between the transmitter and receiver is demonstrated in real time. In contrast, the frames received by the microstrip system are severely distorted (Fig. 5f), even though the test scenario is completely identical. The distortion in the microstrip system is due to the leakage of waves on the discontinuity (Fig. 2b) and the coupling between adjacent microstrips (Fig. 2c). To clearly illustrate the whole process, two movies with almost real-time communications are provided (see Supplementary Information).

## Discussion

This work demonstrates that our design of an SPP-based integrated wireless communication system can overcome the bottleneck of traditional wireless communication. We show that it is possible to achieve line-of-sight real-time wireless communication of subdiffraction-limited EM signals due to the superior properties of the spoof surface plasmon modes, such as the low signal interference between subwavelength channels and the high power sensitivity. On the other hand, the proposed SPP system with planar subwavelength channels can be simply fabricated on commercially available dielectric substrates using printed circuit board (PCB) techniques. We believe that this integrated SPP wireless communication system will open up an avenue to build up future flexible and wearable devices able to efficiently transmit subdiffraction-limited EM signals.

## Materials and methods

### Materials for the dielectric substrate and fabrication

In our design, the dielectric substrate selected is the Rogers RT/duroid 5880, the real and imaginary parts of the relative permittivity *ε*_r_ of which are 2.2 and 0.002, respectively. This is the standard dielectric substrate used for wireless communication applications, as it provides a near-ideal loss-free environment. More detailed information on this material can be found in the datasheet provided by the Rogers Corporation. The system was fabricated in a single layer of dielectric substrate (Rogers RT 5880) with a thickness of 787 μm. A single 18-μm-thick copper layer is printed on the substrate using PCB techniques, which is also used to design passive SPP components of the whole system. Meanwhile, some commercial bare semiconductor chips are used to construct the active SPP components. Specifically, bare semiconductor chips are used to realize the integrated plasmonic active devices as arranged in Fig. 1 and connected to the metawaveguides through bonding wires, which should be regarded as electrical discontinuities.

### The specific geometrical parameters of the metawaveguides

Our metawaveguide inherits the advantages of metamaterials, allowing us to tune the properties of the structure by just changing the geometric parameters. To achieve support wave propagation at 18 GHz (i.e., modulated signal) and suppress wave propagation at 20 GHz (i.e., second harmonic of the LO), the geometric parameters of the metawaveguide are optimized by the commercial software CST Microwave Studio, and the resulting geometrical parameters are listed in Part #1 of Table S1.

### The design of the integrated ultracompact filter

To avoid disturbance caused by the unmodulated LO, a reject band at approximately 10 GHz must be devised. This component should also maintain the linewidth to retain the main advantage of the metawaveguide. Hence, we use for this filtering purpose a metamaterial object, i.e., a split ring resonator (SRR), which is etched on one of the metawaveguide units, as shown in Fig. S3. To optimize its performance, the geometric parameters of the SRR are carefully designed and listed in Part #2 of Table S1.

### The design of the antenna

To provide poor directional radiation, a common patch antenna operating at 18 GHz is also incorporated into our system; the geometric parameters can be obtained according to the method of microwave engineering and the numerical optimization algorithm. The gain and directional diagram associated with its performance can be found in Fig. 2d, and its geometric parameters are listed in Part #3 of Table S1.

### The design of the shared LO

The LO is implemented by the wideband microwave VCO chip ADF5355, which permits a frequency operation bandwidth from 6.8 to 13.6 GHz at one RF output. In this work, the local oscillation is fixed at 10 GHz. Then the local oscillation is amplified by an IF amplifier loaded with the amplifier chip Gali19 and divided into two subwavelength metawaveguides using the power splitter chip EP2C-D+. Details regarding the features and design methods of these chips can be found in the datasheet provided by the company’s website.

### The design of the H-mixer

The H-mixers in our plasmonic device possess two functionalities: the harmonic generating and mixing of frequencies. By integrating the subharmonically pumped MMIC mixer bare chip HMC337 into the metawaveguide, a second-harmonic wave of local oscillation (10 GHz) can be generated and then mixed with the modulated movie signals (1.9 GHz). Hence, RF signals of 18.1 GHz are produced by the H-mixers. Details regarding the features and design method of this chip can be found in the datasheet provided by the company’s website.

### The design of the amplifier chain

The amplifier chains in our SPP-based device are composed of series plasmonic amplifiers designed by integrating amplifier bare chips produced by semiconductor technology into the metawaveguides. The purpose of amplification is not only to compensate for the loss of SPP waves but also to increase the SNR so that the maximum wireless communication distance of this system can be enhanced. In the transmitter, two low-noise amplifiers (LNAs) and one medium-power amplifier (MPA) are employed. In the receiver, two LNAs are employed. The LNA is loaded by the LNA bare chip HMC517 at microwave frequencies (from 17 to 22 GHz) with high gain (approximately 19 dB). The MPA is integrated by the MPA bare chip HMC442 at microwave frequencies (from 17.5 to 21 GHz) with high gain (approximately 14.5 dB). Details regarding the features and design methods of these chips can be found in the datasheet provided by the company’s website.

### High power sensitivity associated with the metawaveguide

Demodulation systems possess a threshold-induced voltage used to recognize the digital signal. Only demodulated signals higher than this threshold will be judged as in the “1” state, which may cause the wrong judgment if signals with the “1” state decay after wireless communication. Hence, by concentrating the field and enhancing the voltage on the metawaveguide, the power sensitivity will be improved compared to conventional microstrips.

### Leaky waves associated with the discontinuity on channels

Connection between the microwave waveguides and chips is realized by bonding wires. In this work, the discontinuity of wave forms may cause serious radiation loss. The radiation can be quantitatively observed by the radiation efficiency. We have found that the radiation efficiency of a microstrip discontinuity is −5.86 dB, while that of a metawaveguide discontinuity is <−10 dB. A narrow gap results in little phase disturbance to the transmission but has obvious near-field distribution discontinuity. Under the same scale, most transmission fields of the microstrip are distributed in the range of approximately 3 h, while most transmission fields of the SPP metawaveguide are distributed in the range of approximately 1.5 h. For the thin bonding gold wire, a tighter field distribution is preferred, which is the reason why the metawaveguide performs much better than the microstrip in addressing the bonding gold wire discontinuity.

### The data rate and bit error rate experiment

In this experiment, first, a pseudorandom digital baseband signal S1 with a set data rate, generated by a bit error rate tester (BERT), is input into the video coding board (detailed information can be found in Supplementary Information) as the extended digital baseband signal source. Then signal S1 is further modulated by the quadrature phase shift keying (QPSK) modulation board (detailed information can be found in Supplementary Information) and enters our plasmonic wireless communication system. The received signal S2 that is obtained by the receiver, QPSK demodulation, and decoding boards will return to the BERT. Through the comparison calculation in the BERT, we obtain the measured bit error rate. Finally, we measure the bit error rates with different data rates to obtain the limited communication speed with an acceptable bit error rate.

## Supplementary information

Supplementary Information

Supplementary Videos

Supplementary Videos

## References

[1] Lerosey G (2007). Focusing beyond the diffraction limit with far-field time reversal. Science.

[2] Wan WJ (2011). Time-reversed lasing and interferometric control of absorption. Science.

[3] Olivieri L (2018). Time-resolved nonlinear ghost imaging. ACS Photonics.

[4] Maire. G (2009). Experimental demonstration of quantitative imaging beyond Abbe’s limit with optical diffraction tomography. Phys. Rev. Lett..

[5] Kim K (2014). High-resolution three-dimensional imaging of red blood cells parasitized by *Plasmodium falciparum* and in situ hemozoin crystals using optical diffraction tomography. J. Biomed. Opt..

[6] Pendry JB (2000). Negative refraction makes a perfect lens. Phys. Rev. Lett..

[7] Engheta N, Ziolkowski RW (2005). A positive future for double-negative metamaterials. IEEE Trans. Microw. Theory Tech..

[8] Yuan GH (2019). Far-field superoscillatory metamaterial superlens. Phys. Rev. Appl..

[9] Lu DL, Liu ZW (2012). Hyperlenses and metalenses for far-field super-resolution imaging. Nat. Commun..

[10] Kaina N (2015). Negative refractive index and acoustic superlens from multiple scattering in single negative metamaterials. Nature.

[11] High AA (2015). Visible-frequency hyperbolic metasurface. Nature.

[12] Pacheco-Peña V (2017). Experimental realization of an epsilon-near-zero graded-index metalens at terahertz frequencies. Phys. Rev. Appl..

[13] Li MY (2016). Eight-port orthogonally dual-polarized antenna array for 5G smartphone applications. IEEE Trans. Antennas Propag..

[14] Gao X (2015). Massive MIMO performance evaluation based on measured propagation data. IEEE Trans. Wirel. Commun..

[15] Ding ZG (2016). The application of MIMO to non-orthogonal multiple access. IEEE Trans. Wirel. Commun..

[16] Barnes WL, Dereux A, Ebbesen TW (2003). Surface plasmon subwavelength optics. Nature.

[17] Shin H, Fan SH (2006). All-angle negative refraction for surface plasmon waves using a metal-dielectric-metal structure. Phys. Rev. Lett..

[18] Pendry JB, Martín-Moreno L, Garcia-Vidal FJ (2004). Mimicking surface plasmons with structured surfaces. Science.

[19] Ayata M (2017). High-speed plasmonic modulator in a single metal layer. Science.

[20] Gao Z (2018). Spoof plasmonics: from metamaterial concept to topological description. Adv. Mater..

[21] Zhang JJ (2017). Spoof plasmon hybridization. Laser Photonics Rev..

[22] Shen XP (2013). Conformal surface plasmons propagating on ultrathin and flexible films. Proc. Natl Acad. Sci. USA.

[23] Gao F (2016). Probing topological protection using a designer surface plasmon structure. Nat. Commun..

[24] Lin X (2017). All-angle negative refraction of highly squeezed plasmon and phonon polaritons in graphene–boron nitride heterostructures. Proc. Natl Acad. Sci. USA.

[25] Gao F (2015). Dispersion-tunable designer-plasmonic resonator with enhanced high-order resonances. Opt. Express.

[26] Lin X (2013). Unidirectional surface plasmons in nonreciprocal graphene. New J. Phys..

[27] Kianinejad A, Chen ZN, Qiu CW (2015). Design and modeling of spoof surface plasmon modes-based microwave slow-wave transmission line. IEEE Trans. Microw. Theory Tech..

[28] Kianinejad A, Chen ZN, Qiu CW (2016). Low-loss spoof surface plasmon slow-wave transmission lines with compact transition and high isolation. IEEE Trans. Microw. Theory Tech..

[29] Luk’yanchuk B (2010). The Fano resonance in plasmonic nanostructures and metamaterials. Nat. Mater..

[30] Maier SA (2001). Plasmonics—a route to nanoscale optical devices. Adv. Mater..

[31] Tame MS (2013). Quantum plasmonics. Nat. Phys..

[32] Ciracì C (2012). Probing the ultimate limits of plasmonic enhancement. Science.

[33] Lin X (2017). Splashing transients of 2D plasmons launched by swift electrons. Sci. Adv..

[34] Xu S (2015). Broadband surface-wave transformation cloak. Proc. Natl Acad. Sci. USA.

[35] Yu NF (2010). Designer spoof surface plasmon structures collimate terahertz laser beams. Nat. Mater..

[36] Pors A (2012). Localized spoof plasmons arise while texturing closed surfaces. Phys. Rev. Lett..

[37] Fernandez-Dominguez AI (2008). Spoof surface plasmon polariton modes propagating along periodically corrugated wires. IEEE J. Sel. Top. Quantum Electron..

[38] Huang XR, Peng RW, Fan RH (2010). Making metals transparent for white light by spoof surface plasmons. Phys. Rev. Lett..

[39] Martin-Cano D (2011). Waveguided spoof surface plasmons with deep-subwavelength lateral confinement. Opt. Lett..

[40] Kim SH (2015). Subwavelength localization and toroidal dipole moment of spoof surface plasmon polaritons. Phys. Rev. B.

[41] Khanikaev AB (2010). One-way extraordinary optical transmission and nonreciprocal spoof plasmons. Phys. Rev. Lett..

[42] Liu XY (2014). Planar surface plasmonic waveguide devices based on symmetric corrugated thin film structures. Opt. Express.

[43] Liu XY (2013). High-order modes of spoof surface plasmonic wave transmission on thin metal film structure. Opt. Express.

[44] Liu LL (2018). Backward phase matching for second harmonic generation in negative-index conformal surface plasmonic metamaterials. Adv. Sci..

[45] Zhang HC (2015). Breaking the challenge of signal integrity using time-domain spoof surface plasmon polaritons. ACS Photonics.

[46] Liang Y (2015). On-chip sub-terahertz surface plasmon polariton transmission lines in CMOS. Sci. Rep..

[47] Gao XX (2019). Crosstalk suppression based on mode mismatch between spoof SPP transmission line and microstrip. IEEE Trans. Compon. Packaging Manuf. Technol..

