# Supplementary material for: A plasmonic route for the integrated wireless communication of subdiffraction-limited signals
Source: Light Sci Appl. 2020 Jul 2;9:113. doi: 10.1038/s41377-020-00355-y (PMC7329838; doi:10.1038/s41377-020-00355-y)
Supplement: Supplementary file 1 — Supplementary Information [file 41377_2020_355_MOESM1_ESM.docx]

Supplementary Information for

**A plasmonic route for the integrated wireless communication of subdiffraction-limited signals**

Hao Chi Zhang^1^, Le Peng Zhang^1^, Pei Hang He^1^, Jie Xu^1^, Cheng Qian^1^, Francisco J. Garcia-Vidal^2,3*^, and Tie Jun Cui^1*^

^1^*State Key Laboratory of Millimeter Waves, Southeast University, Nanjing 210096, China.*

^2^*Departamento de Física Teórica de la Materia Condensada and Condensed Matter Physics Center (IFIMAC), Universidad Autónoma de Madrid, Madrid, Spain.*

^3^*Donostia International Physics Center (DIPC), Donostia/San Sebastian, Spain.*

* Email: [fj.garcia@uam.es](mailto:fj.garcia@uam.es) or [tjcui@seu.edu.cn](mailto:tjcui@seu.edu.cn)

**METHODS**

**Test environment of the wireless communication systems.** The purpose of the experiment is to verify the transmission effect of plasmonic wireless communication system under over line-of-sight conditions. The experiment was completed at our laboratory of Southeast university in Nanjing, China. Plasmonic wireless communication system consists of transmitting section and receiving section, which are located inside and outside the laboratory room separately. The transmitting section is located in the corridor outside the room and the receiving section located in the room. The laboratory room door is open. The two are completely separated by one concrete wall and placed close to the inside and outside of the wall. Electromagnetic waves cannot travel through the wall, which satisfies the over line-of-sight condition fully. Other measuring equipment (such as vector network analyzer), tables and chairs that are not related to this experiment are also placed in the room where the receiving section is placed. The corridor where the transmitting section is placed communicates with the open environment (please see the movie S1). In short, the experimental environment is complex and can represent the general environment to some extent.

In order to further verify the transmission effect of plasmonic wireless communication system, the experiment of maximum transmission distance under line-of-sight conditions was carried out at another laboratory, as shown in Fig. S1. The components of the line-of-sight test system are exactly the same as the one under over line-of-sight conditions. The difference is that the system are in the same room and placed at a certain distance, fully meeting the condition of line-of-sight. Other measuring equipment (such as vector network analyzer), tables and chairs that are not related to this experiment are also placed in this room, and the door of the room is open, which implies the spatial EM wave will affect the system.

**Experimental video.** The video shows the transmission experiment of the plasmonic wireless communication system and microstrip wireless communication system under over line-of-sight transmission condition.

First, the plasmonic wireless communication system was tested, and the experimental process fully met the over line-of-sight transmission conditions. As shown in the video, the transmitter and receiver are separately placed inside and outside the laboratory wall, and the height of antenna platforms were adjusted to make sure that the receiving and transmitting antennas are at the same height. Two coded modulation boards on the transmitter are connected to two laptops which act as the sources of two different high-definition movies (‘*The Wandering Earth*’ and ‘*Titanic*’). The transmitter is powered by a voltage source (Agilent U8001A) at a voltage of 6.05V. At the receiver, the decoding demodulation boards were connected to another two laptops. The receiver is powered by a voltage source (KEYSIGHT E3631A) of 5.99V. As shown in the video, two different movies are played on the two screens and both films played smoothly and clearly, which proves that the interference between two signals in our plasmonic wireless system is very small. The measured eye pattern and constellation map of the signals in plasmonic wireless communication system under over line-of-sight transmission condition is shown in Fig. 4 d and e. Besides, the measured frequency spectrum of signals from different components of the plasmonic wireless communication system under over line-of-sight transmission conditions are shown in Fig. S2.

After that, the microstrip wireless communication system was tested, and the experimental process is identical to that of the plasmonic system. In the same way, two coded modulation boards on the transmitter end are connected to the same movie sources. At the receiver, the decoding demodulation boards were connected to two laptops. However, the two screens were playing the same movie very intermittently, and the communication quality of the case where only one channel is employed is much better than that of the case where both the channels are employed, which proves that the interference between two video signals in microstrip wireless communication system is very serious.

**Video processing module.** The whole video processing module is divided into two parts: transmitting section and receiving section. The transmitting section includes video coding board and QPSK modulation board and the receiving section includes video decoding board and QPSK demodulation board.

**Video coding board.** The main function of the video coding board is to realize h. 264 compression coding and it can support up to 1080P@60fps, downward compatible with other resolutions and frame rate. The board can support mini HDMI audio and video input interface and video input standard supports various modes such as 1080 60P, 1080 50P, etc. The coding board is connected with the back-end QPSK modulation board via the FPC Connector to complete the transmission of video code stream. In addition, the control serial port can be used for board parameter configuration (such as frame rate, etc.). It also supports Micro USB interface for software writing, upgrading and maintenance. The power supply voltage of the board is 6-13v, with special power input interface.

**QPSK modulation board.** The QPSK modulation board is connected to the video coding board via the FPC Main interface to receive digital video streams. Then the modulated RF signal is output through IPEX RF interface. QPSK modulation parameters can be set through the FPC_CON interface or the FPC_Main interface. The working frequency band of the modulation board is from 70MHz to 6GHz (the default setting is 300MHz). The board can be configured with convolution encoding ratio of 1/2, 2/3, 3/4, 5/6, 7/8 and supports QPSK, QAM16, QAM64. The configurable bandwidth is from 2MHz to 8MHz with 1 KHz step. RF output power can be configured as from -5dbm to 94dbm and the supply voltage is from 5.5 to 16V.The size of board is 50mm×45mm. There are two LED indicators on the board: the left indicator is flashing to indicate the emission data, and the right indicator is the power indicator.

Video coding board is stacked with QPSK modulation board via FPC Connector to form video coding and modulation transmitting module. The Config. UART interface on coding board can be externally connected to the serial port control panel to set operating parameters, such as frame rate, working frequency band, wireless bandwidth, transmitting power, etc.

**QPSK demodulation board.** QPSK demodulation receiving board is used to achieve QPSK signal reception demodulation function. The receiving frequency band is from 70MHz to 6000MHz (the default setting is 300MHz). The receiving sensitivity is -97±1dbm (8MHz) and -98±1dbm (6MHz). The supported bandwidth is from 3.2 MHz to 8MHz with 0.1MHz step. The modulation modes are QPSK, QAM16 and QAM64 and the power consumption is 1.3w and a power indicator is installed on the board.

**Video decoding board.** Video decoding board card can achieve H. 264 video decompression playback function and it can support up to 1080P@60fps, downward compatible with other resolutions and frame rate. The board is equipped with HDMI-A audio and video output port. The decoding board also has Config. UART, which can be connected to PC or parameter control panel for parameter configuration. The decoding board supports external USB disk or mobile hard disk and the TF card (used for software upgrade and maintenance, data storage, etc.) can be also supported. The board is equipped with three LED lights: power indicator, RF signal receiving indicator and recording indicator. The keys on the board are OSD switch and record switch respectively. Power supply voltage of this board is from 6V to15V.

The video decoding board and QPSK demodulation board are stacked together to form a demodulation and decoding receiver to realize QPSK signal receiving/demodulation and video decoding output.

**Acknowledgments** This work has been funded by the National Key Research and Development Program of China (Grant Nos. 2017YFA0700201, 2017YFA0700202 and 2017YFA0700203), by the National Natural Science Foundation of China (Grant Nos. 61571117, 61631007, 61701108, and 61871127), and by the 111 Project (Grant No. 111-2-05). F.J.G.-V. acknowledges support by the QuantERA program of the European Commission with funding by the Spanish AEI through project PCI2018-093145, and by the “Maria de Maeztu” programme for Units of Excellence in R&D (MDM-2014-0377).

**Author contributions** All authors contributed extensively to this work, including the analysis, discussion, and interpretation of the results. All authors discussed, revised, and approved the manuscript.

**Competing interests** Authors declare no competing interests.


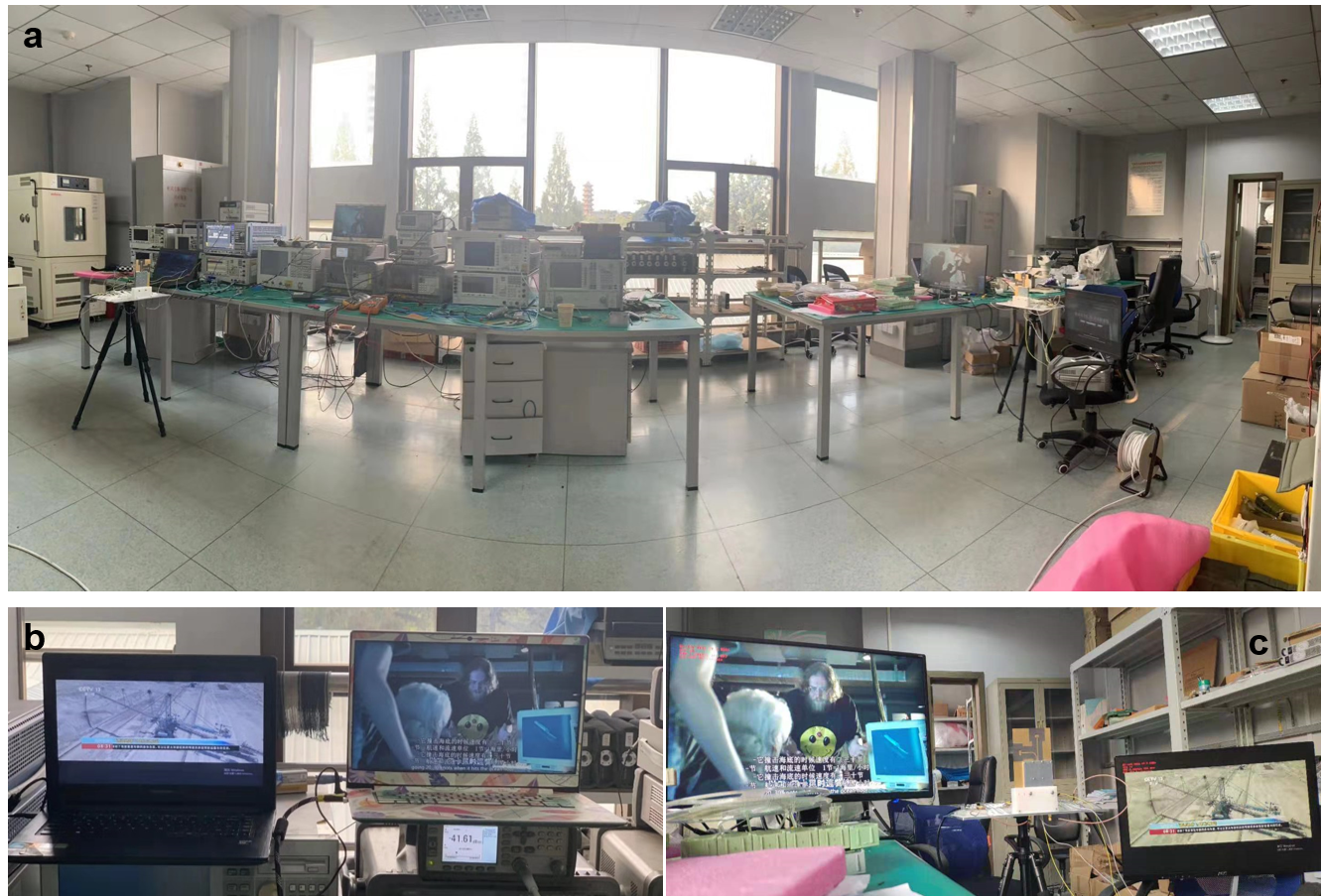


**Fig. S1 The experiment of line-of-sight transmission of two movies (‘The Wandering Earth’ and ‘Titanic’), which are transmitted in the two channels.**  **a**, Test scenario schematic of line-of-sight channel, where two 20 dB attenuators are added to both communication channels to mimic an 800 m long-distance wireless communication (The transmitting antenna and receiving antenna are about 8 meters apart). **b** and **c**, Photographs of transmitter screens (b) and receiver screens (c) at same time. In this experiment, two movies are transmitted in the two channels, respectively. As expected, the two movies transmission on the receiver is clear.


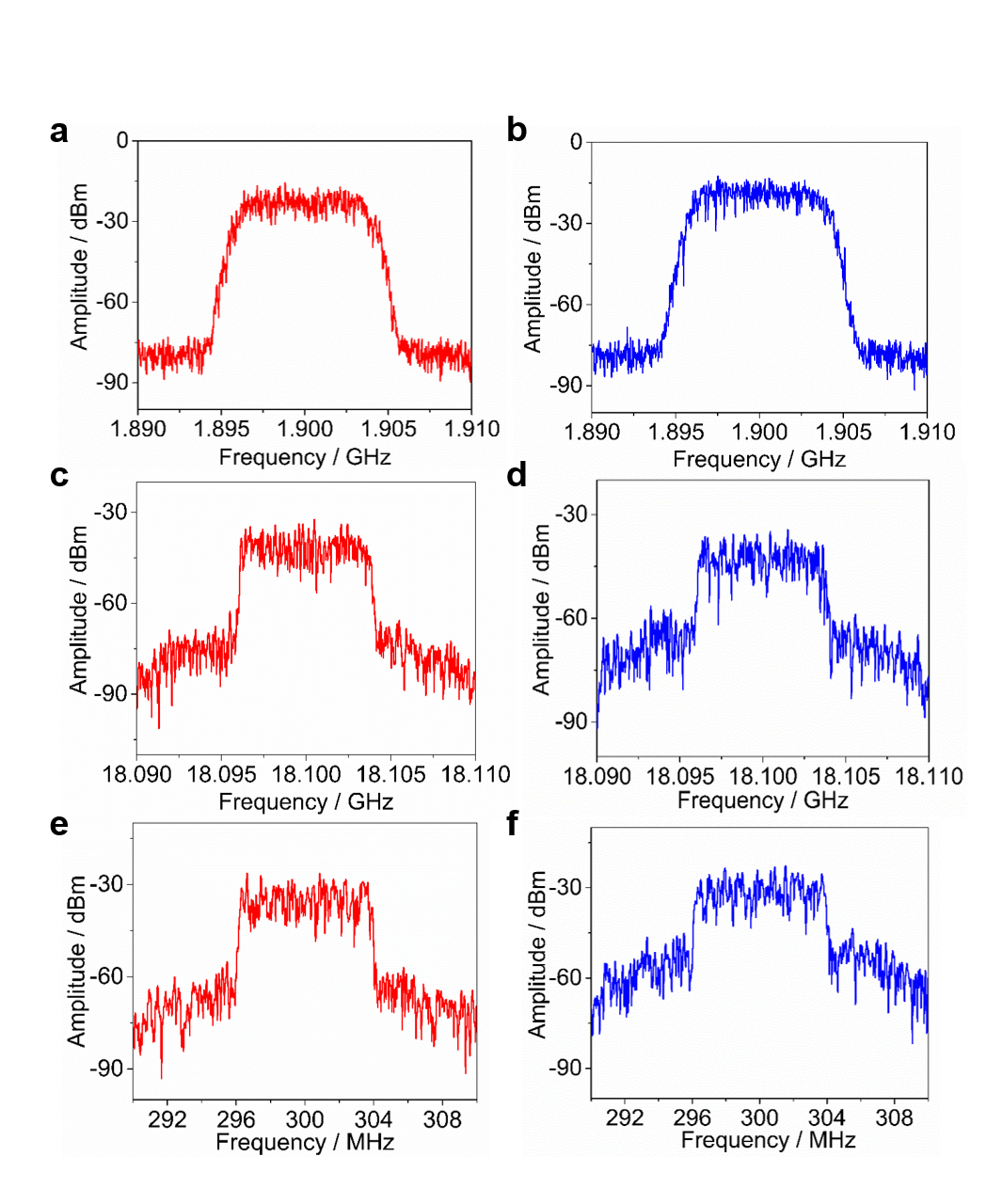


**Fig. S2 The measured frequency spectrum of signals from different components of the plasmonic wireless communication system under over line-of-sight transmission conditions.** **a** and **b**, The coded signal from the transmitter in channel #1 (**a**) and channel #2 (**b**). **c** and **d**, the radiofrequency signals emitted by antennas #1 (**c**) and antennas #2 (**d**). **e** and **f**, the input signal of the receiver in channel #1 (**e**) and channel #2 (**f**).

**
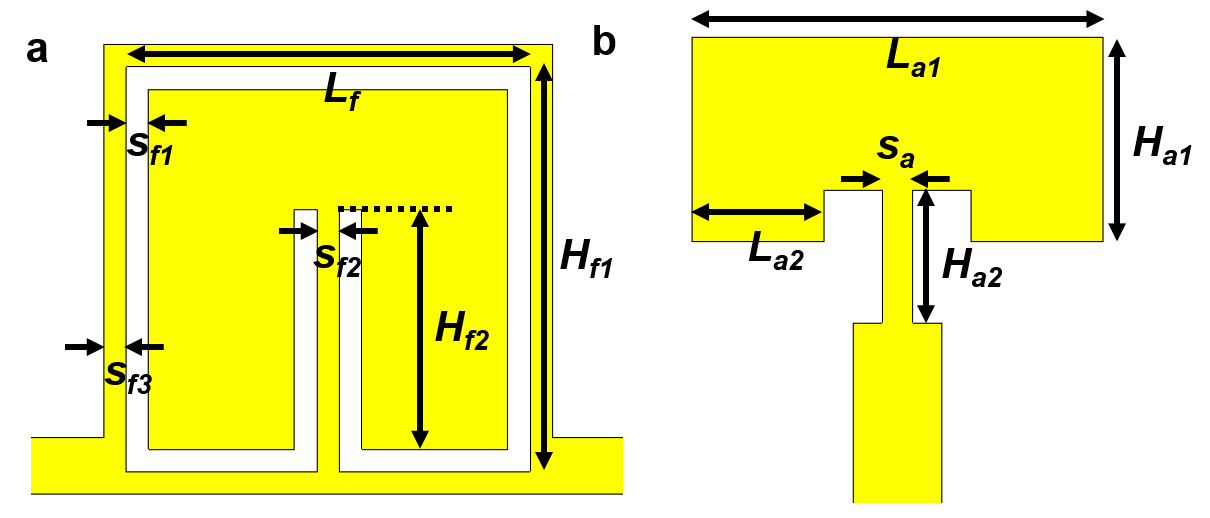
**

**Fig. S3 The geometrical structures of the ultra-compact filter (a) and the antenna (b).**

**
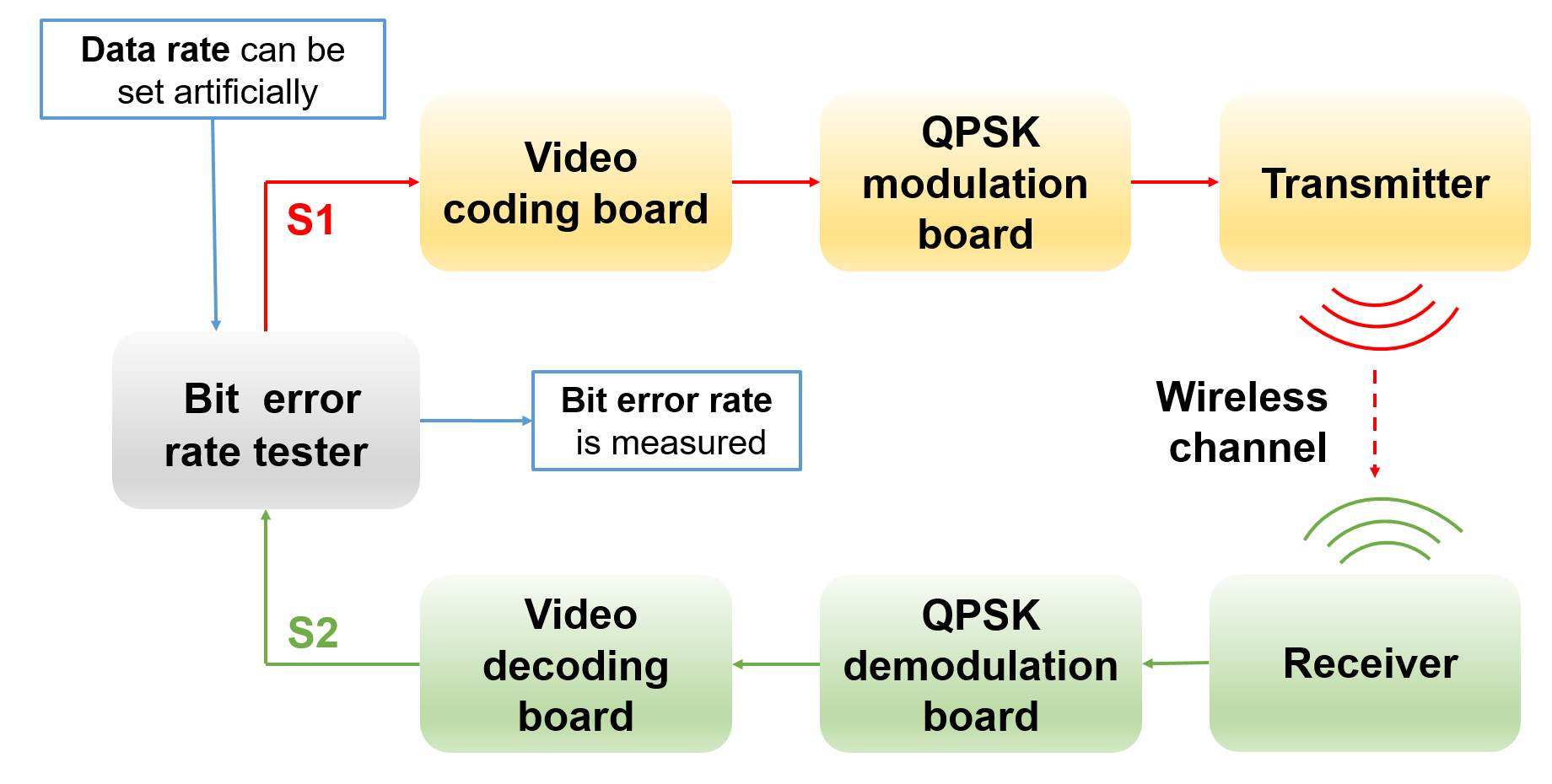
**

**Fig. S4 Test schematic of bit error rate and transmission speed.**

**Table S1. The geometric parameters of the plasmonic system**

| #1: The geometric parameters of the metawaveguide | | | |
| --- | --- | --- | --- |
| Period of unit *p* | 2.8 mm | Width of strip *h* | 2.4 mm |
| Width of split *a* | 0.4 mm | High of split *d* | 2.1 mm |
| #2: The geometric parameters of SRR | | | |
| Length of SRR *L_f_* | 2.16 mm | Height of SRR *H_f1_* | 2.16 mm |
| Line width of SRR *S_f1_* | 0.12 mm | Width of split *S_f2_* | 0.12 mm |
| Height of split *H_f2_* | 1.28 mm | SPP-SRR Gap | 0.15 mm |
| #3: The geometric parameters of antenna | | | |
| Length of antenna *L_a1_* | 11.1 mm | Width of antenna *H_a1_* | 5.55 mm |
| Length of extension *L_a2_* | 3.55 mm | Length of transition *H_a2_* | 3.59 mm |
| Width of transition *S_a_* | 0.84 mm |  |  |

**Supplementary Videos. The videos of the process of over line-of-sight transmission of subdiffraction-limited electromagnetic signals in a complex environment.** In the transmitter, two movies (‘*The Wandering Earth*’ and ‘*Titanic*’) are transmitted in the two channels, respectively. The transmitter and receiver are separated by a concrete wall, which cannot be penetrated by electromagnetic waves at this frequency range. In the receiver of the plasmonic wireless communication system (please see the movie S1), two films play smoothly and clearly. On the contrary, in the receiver of the microstrip wireless communication system (please see the movie S2), two films display badly.
